# Resting EEG in psychosis and at-risk populations — A possible endophenotype?

**DOI:** 10.1016/j.schres.2013.12.017

**Published:** 2014-03

**Authors:** Siri Ranlund, Judith Nottage, Madiha Shaikh, Anirban Dutt, Miguel Constante, Muriel Walshe, Mei-Hua Hall, Karl Friston, Robin Murray, Elvira Bramon

**Affiliations:** a Mental Health Sciences Unit & Institute of Cognitive Neuroscience, University College London, W1W 7EJ, United Kingdom; b NIHR Biomedical Research Centre for Mental Health at the South London and Maudsley NHS Foundation Trust and Institute of Psychiatry, Kings College London, WC2R 2LS, United Kingdom; c Department of Psychology, Royal Holloway, University of London, TW20 0EX, United Kingdom; d Psychiatry Department, Hospital Beatriz Ângelo, 2674-514 Loures, Lisbon, Portugal; e Psychology Research Laboratory, Harvard Medical School, McLean Hospital, Belmont, MA 02478, USA; f The Wellcome Trust Centre for Neuroimaging, University College London, WC1N 3BG, United Kingdom

**Keywords:** Endophenotype, Schizophrenia, Psychosis, Resting EEG, At risk mental state, Electroencephalogram

## Abstract

**Background:**

Finding reliable endophenotypes for psychosis could lead to an improved understanding of aetiology, and provide useful alternative phenotypes for genetic association studies. Resting quantitative electroencephalography (QEEG) activity has been shown to be heritable and reliable over time. However, QEEG research in patients with psychosis has shown inconsistent and even contradictory findings, and studies of at-risk populations are scarce. Hence, this study aimed to investigate whether resting QEEG activity represents a candidate endophenotype for psychosis.

**Method:**

QEEG activity at rest was compared in four frequency bands (delta, theta, alpha, and beta), between chronic patients with psychosis (N = 48), first episode patients (N = 46), at-risk populations (“at risk mental state”, N = 33; healthy relatives of patients, N = 45), and healthy controls (N = 107).

**Results:**

Results showed that chronic patients had significantly increased resting QEEG amplitudes in delta and theta frequencies compared to healthy controls. However, first episode patients and at-risk populations did not differ from controls in these frequency bands. There were no group differences in alpha or beta frequency bands.

**Conclusion:**

Since no abnormalities were found in first episode patients, ARMS, or healthy relatives, resting QEEG activity in the frequency bands examined is unlikely to be related to genetic predisposition to psychosis. Rather than endophenotypes, the low frequency abnormalities observed in chronic patients are probably related to illness progression and/or to the long-term effects of treatments.

## 1 Introduction

Although psychotic disorders are highly heritable (Cardno et al., 1999; Lichtenstein et al., 2009; Rijsdijk et al., 2011; Fowler et al., 2012) understanding their specific genetic causes has proven harder than anticipated (Hardy et al., 2008). It is only recently that the first genetic risk factors have been identified through large international collaborative efforts; however, little is known about the role of the associated loci (Ripke et al., 2011; Sklar et al., 2011; Rees et al., 2012; Sullivan et al., 2012; Smoller et al., 2013). The use of endophenotypes, which are heritable biological markers characterising the disease (Gottesman and Gould, 2003), could lead to a better understanding of the mechanisms through which variation in these genes leads to the disease (Braff et al., 2007; Hall and Smoller, 2010; Glahn et al., 2012).

Electroencephalography (EEG) measures ongoing electrical brain activity, and provides a possible basis for endophenotypes of brain function associated with psychosis (Blackwood et al., 2001; Sumich et al., 2006; Hall et al., 2011). Several such measures are highly heritable (Tang et al., 2007; Zietsch et al., 2007; Boutros et al., 2008) and some event-related potentials have been shown to be promising endophenotypes for psychotic disorders (Bramon et al., 2005; Schulze et al., 2008; Turetsky et al., 2008; Decoster et al., 2012; Shaikh et al., 2013). This study focused on quantitative EEG (QEEG) at rest, where psychiatric patients have shown abnormal patterns of activity compared to healthy controls (Hughes and John, 1999; Coburn et al., 2006; Boutros et al., 2008).

Relatively little research has been conducted on resting QEEG activity in patients with psychosis, especially in populations at-risk for the illness, and results have been inconsistent and sometimes even contradictory (Gross et al., 2006; Boutros et al., 2008). Nonetheless, psychotic patients generally exhibit increased slow wave QEEG activity in the delta (1–4 Hz) and theta (4–8 Hz) bands (Sponheim et al., 1994; Sponheim et al., 2000; Winterer et al., 2001; Kirino, 2004; Harris et al., 2006; Begic et al., 2011; Hong et al., 2012), and decreased alpha (8–13 Hz) activity (Sponheim et al., 2003; Harris et al., 2006; Begic et al., 2011). In terms of resting beta (13–21 Hz) activity, results are inconsistent, with studies reporting both decreased (John et al., 1994) and increased (Wuebben and Winterer, 2001; Begic et al., 2011) activity, as well as no abnormalities in patients with psychosis (Sponheim et al., 1994; Winterer et al., 2001; Mientus et al., 2002; Hong et al., 2012). It is therefore unclear whether resting QEEG represents a useful endophenotype for psychosis, which speaks to the need for further research in this area.

The aim of this study was to investigate the role resting QEEG abnormalities play in the aetiology of psychosis, and whether it can provide an endophenotype for the illness. Quantitative EEG amplitudes at rest were compared across four frequency bands, between five groups; chronic psychotic patients, first episode patients, individuals at-risk of developing psychosis, unaffected relatives of patients, and healthy controls. Psychosis was broadly defined, including patients with schizophrenia, bipolar disorder (with a history of psychotic symptoms), schizoaffective disorder, as well as other psychotic illnesses. Based on past findings, it was hypothesised that amplitudes in delta and theta frequency bands would be increased, and amplitude in the alpha band would be reduced, in patients with psychosis as well as in populations at risk, compared to healthy controls. In the beta frequency band, no direction of abnormalities was predicted. Impairments were predicted to be most severe in the patients.

## 2 Method and materials

### 2.1 Sample and clinical assessments

The total sample of 279 participants was recruited from the South London and Maudsley NHS Foundation Trust (including “Outreach and Support in South London” and the Lambeth Early Psychosis Intervention service), as well as through collaboration with the charity Re-Think (www.rethink.org), and advertisements in the local and national media.

All participants were clinically interviewed to confirm or exclude a Diagnostic and Statistical Manual of Mental Disorders, Fourth Edition (DSM-IV) (APA, 1994) diagnosis. The interview instruments used were the Structured Clinical Interview for DSM Disorders (SCID) (First et al., 1995) or the Schedule of Affective Disorders and Schizophrenia Lifetime Version (SADS-L) (Endicott and Spitzer, 1978), and the Positive and Negative Syndrome Scale (PANSS) (Kay et al., 1987). Information regarding psychiatric diagnoses of family members not directly assessed was collected from the most reliable informant(s) with the Family Interview for Genetic Studies (Maxwell, 1992). Additional information was collected from medical notes where available. Participants were excluded if they had a diagnosis of alcohol or substance dependence in the 12 months preceding study entry, any neurological disorders, or head injury with loss of consciousness for more than a few minutes.

The total sample included five groups. At the time of testing, chronic patients (N = 48) had been ill for more than three years, and first episode patients (N = 46) less than three years. The cut-off of 3 years reflects the maximum amount of time our local Early Intervention Service – where the first episode patients were recruited from – followed up their patients. This is comparable to other early psychosis research (Singh et al., 2011; Saleem et al., 2013). A full breakdown of the diagnoses in these two patient groups can be found in Table 1. Individuals with an “at risk mental state” (ARMS, N = 33) were assessed using criteria in the Comprehensive Assessment for At Risk Mental State (CAARMS) (Yung et al., 2005; Morrison et al., 2006).

Healthy first-degree relatives of chronic patients (N = 45) had no personal history of any psychosis spectrum illness. Healthy controls (N = 107) had no personal or family history of any psychotic disorders. Having a personal history of other non-psychotic psychiatric illnesses did not constitute an exclusion criteria for relatives or controls, provided they were well and not taking any psychotropic medication at the time of testing and for the preceding 12 months. This was to avoid recruiting biased control groups, unrepresentative of the local population.

After a complete description of the study, all participants gave their written informed consent. The study was approved by the Research Ethics Committee at the Institute of Psychiatry, King's College London.

### 2.2 EEG data acquisition

Resting EEG data was collected using either a 64-channel Synamps or a 40-channel Nuamps amplifier and respectively 64 or 40 channel quick caps with sintered silver/silver-chloride electrodes, placed according to the International 10/20 system (Jasper, 1958). All data was continuously digitised at 1000 Hz, with a 0–200 Hz band-pass filter. Electrode impedances were kept below 5 kΩ (Bramon et al., 2008; Shaikh et al., 2013).

For EEG data collected from 40 channels, unipolar electrodes placed on the outer canthi of both eyes, and above and below the left eye monitored eye movements. Linked ear lobes served as reference, and FPZ was the ground (Frangou et al., 1997). For EEG data collected using 64 channels, bipolar vertical and horizontal electro-oculographs monitored eye movements. Bilateral mastoids served as reference, and AFZ was the ground (Bramon et al., 2008; Shaikh et al., 2012).

EEG recordings were collected in a quiet room with participants sitting down comfortably. They were asked to keep their eyes closed for 20 s and then open for 20 s, during a total of 5 min. Resting EEG data collection was followed by other EEG procedures reported elsewhere (e.g. Schulze et al., 2008; Shaikh et al., 2011; Dutt et al., 2012).

### 2.3 Data processing

Signal processing was conducted using Neuroscan 4.3 software (www.neuroscan.com) and MATLAB (www.mathworks.co.uk). Sequential epochs of 2048 ms were created from the continuous EEG files, separately for eyes-open and eyes-closed conditions. Automatic artefact detection rejected sweeps with activity exceeding ± 100 μV (Reinhart et al., 2011). EEG amplitude (μV) was calculated using the Fast Fourier Transformation using a Hanning window with 10% taper length. Only the EEG segments required under eyes-closed conditions were included in further statistical analyses — to suppress the effect of ocular artefacts (Zimmermann et al., 2010; Lavoie et al., 2012). After artefact rejection and exclusion of eyes open data, on average 101 s remained per subject for analysis (mean = 101.20, SD = 29.33). This did not differ between groups.

Amplitude was analysed for four individual segments of the EEG spectrum; delta (1.95–3.90 Hz), theta (4.39–7.32 Hz), alpha (8.30–12.70 Hz), and beta (13.20–21.00 Hz). These frequency bands are typical of similar research (Boutros et al., 2008), except that we chose not to analyse frequencies above 21 Hz. This was due to accumulating evidence that frequencies above 21 Hz can still be substantially contaminated by scalp electromyogram activity (EMG), even after rejection of large EMG bursts (Whitham et al., 2007; Shackman et al., 2010; Nottage et al., 2013).

For data-reduction purposes (to minimize type I error), only the three midline EEG channels, frontal (FZ), central (CZ), and parietal (PZ), were chosen for statistical analysis (Harris et al., 2006).

### 2.4 Statistical analysis

Mixed effects linear regression models were used to examine EEG amplitude, separately for each frequency band, with fixed effects of clinical group and scalp site, and random effects of family and subject. Hence, correlations between members of the same family were modelled, to maintain correct type-1 error rates. The dependent variable was EEG amplitude in μV at each of the four frequency bands (delta, theta, alpha, and beta). The independent variables were participant group — a between-subjects variable with five levels (chronic patients, first episode patients, ARMS, relatives, and controls), and region — a within-subjects variable with three levels (FZ, CZ, and PZ). Age and gender were controlled for (as nuisance regressors) in all analyses. Since EEG data were collected using two different laboratories, due to an upgrade of the EEG equipment, this was also controlled for; by including a binary regressor in the analysis. The control group and FZ were used as reference categories in all inferential tests.

A Bonferroni correction for four tests (delta, theta, alpha, and beta frequency bands) was applied, with the significance threshold thus set to p = 0.05/4 = 0.0125. Statistical analyses were performed using STATA version 11.2 (www.stata.com) and SPSS version 17.1 (www.spss.com).

## 3 Results

### 3.1 Sample characteristics

Demographic data for the entire sample is provided in Table 1. T-tests showed that each group differed significantly from the control group in mean age, with the chronic patients and relatives being older (both groups p < 0.001), and the first episodes and at-risk mental state (ARMS) individuals being younger (p < 0.001) than controls. Chi square tests indicated that there were significantly more males in the first episode group in comparison to the control group (p = 0.05). No other group differed in gender distribution compared to controls. To control for any age or gender effects on the resting EEG, we included these effects as covariates in all analyses. As described in Table 1, the majority of chronic and first episode patients were taking antipsychotics at the time of testing, whereas the relatives, ARMS and controls were free of any psychotropic medication at the time of testing.

The mean EEG amplitudes (μV) for each group, in the four frequency bands, are shown in Table 2. All EEG outcome measures were log-transformed (log10 + 1) to ensure normality of random effects. Correlations between EEG amplitude in the four frequency bands and the three scalp sites were all significant, with correlation coefficients ranging between 0.21 and 0.99 (see Supplementary material). Nevertheless, we adjusted all our analyses for multiple testing (4 tests).

Most participants (first episodes, ARMS, and controls) were recruited individually, but the chronic patients and their relatives were recruited as part of a family study. Of the 279 participants, 174 (62.37%) were singletons, 72 (25.81%) were part of families with two members in the study, 21 (7.53%) were in three-person families, and 12 (4.30%) were part of families with four members participating.

### 3.2 Mixed effects linear regression

Four mixed effects linear regression models were analysed. In the delta band, chronic patients had on average 0.208 μV greater amplitude than controls, which was statistically significant (p < 0.001; Est. diff: 0.082 log μV; 95% CI 0.046–0.118 log μV). No other group differed significantly from the control group in resting delta EEG amplitude.

In the theta frequency band chronic patients had significantly greater resting amplitude compared to controls (p < 0.001; Est. diff: 0.136 log μV; 95% CI 0.083–0.190 log μV), with a 0.368 μV average increase in amplitude. No other group differed significantly from the controls in resting theta activity

In the alpha and beta frequency bands, the control group did not differ significantly from any other group in resting EEG amplitude.

**Fig. 1:**
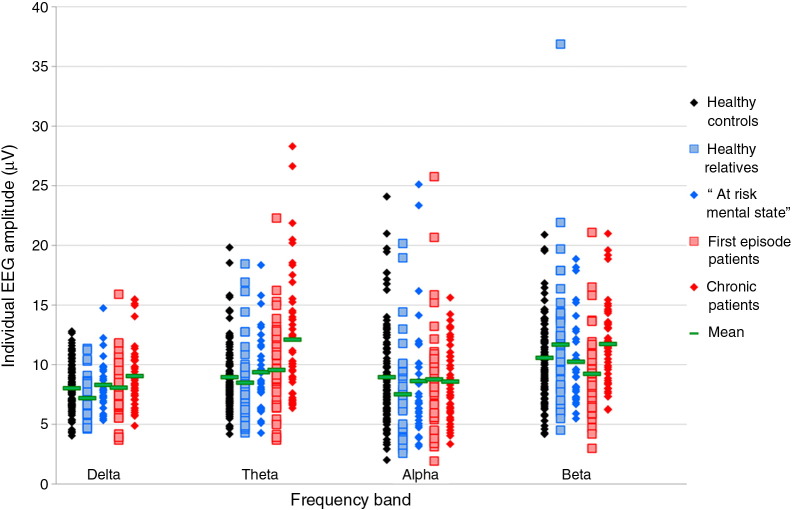
Resting EEG amplitude (μV) in the four frequency bands and the five participant groups, uncorrected for covariates.

Fig. 1 shows EEG amplitudes (μV) across the five groups, for all four frequency bands. Full details of these results, including main effects of covariates, can be found in the Supplementary material. Importantly, the effect of the two different EEG laboratories used for data collection was not significant in any frequency band, justifying pooling the two datasets in one analysis.

Since a broad definition of psychosis was used in this study, the analyses were repeated using a narrow definition of schizophrenia and schizophreniform psychosis, to investigate whether this would affect the results. We excluded all patients with a diagnosis of schizoaffective disorder, brief psychotic disorder, bipolar I disorder, and psychotic disorder not otherwise specified (15 chronic and 8 first episode patients), as well as their relatives (14). These analyses led to results very similar to those using the full dataset, and have not been reported further.

To further investigate potential differences in resting EEG between the groups, we repeated the 4 regression models post-hoc, using the chronic patient group as the reference category. These results are presented in the Supplementary material for the interested reader.

## 4 Discussion

The aim of the current study was to compare quantitative EEG (QEEG) activity at rest in four frequency bands, in patients with psychosis, two populations at-risk of the disease, and healthy controls, to investigate whether QEEG could be used as possible endophenotypes for the illness. The main significant findings are summarised in Table 3.

Our a-priori hypotheses were partly supported; chronic patients showed significantly increased resting delta and theta activity compared to healthy controls. However, first episode patients, individuals with an at-risk mental state (ARMS), and relatives of chronic patients did not differ from controls in these frequencies. Furthermore, there were no significant group differences in resting alpha or beta QEEG activity.

Increased slow wave resting QEEG activity in delta and theta bands in chronic patients with psychosis appears to be fairly well replicated across studies (Sponheim et al., 1994; Omori et al., 1995; Sponheim et al., 2000; Winterer et al., 2001; Kirino, 2004; Harris et al., 2006; Begic et al., 2011; Hong et al., 2012), and supported by our current results. However, our study did not find any significant differences in delta or theta resting activity between the control group and first episode patients or at-risk populations (including both clinically at-risk and genetically predisposed groups). Previous studies on such groups are limited, with inconclusive findings. Abnormalities similar to chronic patients have been observed in first episode patients (Clementz et al., 1994; Sponheim et al., 1994), ARMS (Gschwandtner et al., 2009) and healthy controls (Alfimova and Uvarova, 2003), but several studies have also failed to show abnormalities in these populations (Winterer et al., 2001; Wuebben and Winterer, 2001; Harris et al., 2006). John et al. (1994) found, similarly to our results, that chronic but not first episode schizophrenic patients had increased delta and theta resting activity.

In comparison to the slower frequencies, less research has been conducted on resting alpha QEEG activity in psychosis. As in our study, Mientus et al. (2002) reported no evidence of alpha impairments in patients. However, several previous studies on resting alpha have found a decrease in activity in psychotic patients compared to healthy controls (Omori et al., 1995; Sponheim et al., 2003; Harris et al., 2006; Begic et al., 2011).

In the beta frequency band, we did not find any significant group differences in resting QEEG activity. However, a slight increase of activity was observed in chronic patients compared to controls (not reaching significance after correction for multiple testing), and post-hoc comparison between chronic and first episode patients revealed an increase of beta activity in the former group at a trend level (see Supplementary material). Together this might indicate an abnormality in chronic psychotic patients, although more research is needed to confirm if this is the case. The literature on resting beta activity in psychosis is inconsistent, with several studies reporting no resting beta abnormalities in psychotic patients (Sponheim et al., 1994; Winterer et al., 2001; Mientus et al., 2002; Hong et al., 2012), although both decreased (John et al., 1994) and increased (Wuebben and Winterer, 2001; Begic et al., 2011) activity has also been observed. Finally, our study did not find any differences in beta amplitude between controls and first episode patients or at-risk populations. Past research on such populations has also largely failed to find significant impairments in these groups (Sponheim et al., 1994; Winterer et al., 2001; Harris et al., 2006; Hong et al., 2012).

Taken together, the current results did not show any statistically significant differences in resting QEEG activity of any frequency band between controls and first episode patients or at-risk populations, including ARMS and unaffected relatives of psychotic patients. This indicates, as also argued by Winterer et al. (2001), that low frequency QEEG abnormalities seen in chronic psychotic patients are likely related to the illness process, or to long-term effects of treatments, rather than to genetic risk for the disorder. Hence, resting QEEG activity (of the four frequency bands examined) does not appear to be promising candidate endophenotypes for genetic research in psychosis.

Nevertheless, low frequency resting QEEG abnormalities, in the delta and theta bands, were observed in chronic psychotic patients compared to healthy controls. This could be a useful biomarker in non-genetic research, perhaps investigating chronicity of the illness or cognitive deficits characterising psychosis, which are often associated with an enduring illness (Hyman and Fenton, 2003; Insel, 2010), or research into prediction of medication-responses. More research is needed to investigate this.

From an aetiological perspective, our findings of increased low-frequency activity (and previous reports of similar abnormalities) are consistent with recent theoretical treatments of psychosis as false perceptual inference (Fletcher and Frith, 2009; Adams et al., 2013). In this formulation, acute psychotic symptoms are regarded as a compensation for a failure of sensory attenuation. In other words, psychotic symptoms arise due to assigning too much salience or precision to high level representations to compensate for precise sensory (low level) inputs (c.f., aberrant salience; Howes and Kapur, 2009). In this setting, negative symptoms or chronic states are seen as a decompensation, with a relative loss of precision at higher levels of the neuronal hierarchy. In this context, precision corresponds to the post-synaptic gain of pyramidal cells reporting prediction errors in hierarchical predictive coding (Bastos Andre et al., 2012; Adams et al., 2013). This is important because a decrease in postsynaptic gain or efficacy leads to a preponderance of lower frequencies relative to higher frequencies in endogenous or resting state activity (Kilner et al., 2005). In short, our chronic group may be evidencing reduced synaptic gain at higher hierarchical levels and a shift in the characteristic frequencies of neuronal fluctuations to lower frequencies. Whether this is a primary aetiological factor, a characteristic part of the disease process, or a response to medication remains an open question.

Importantly, since antipsychotic drugs cross the blood–brain barrier and influence many parameters of brain function (e.g. Joutsiniemi et al., 2001; Knott et al., 2001), it is possible that these medications contribute or lead to resting QEEG abnormalities observed in psychotic patients. This could be an important confounder in our current findings, suggesting that true illness-related effects on resting EEG are nuanced by medication. However, it has also been argued that antipsychotics are unlikely to account for QEEG abnormalities seen in chronic patients, since such alterations have also been found in unmedicated patients (Omori et al., 1995; Merrin and Floyd, 1996; Wuebben and Winterer, 2001; Boutros et al., 2008). Since both the chronic and the first episode patient groups were medicated in the current sample, medication effects alone do not appear to fully explain why no abnormalities were observed in the latter group. Nevertheless, the effects of antipsychotic drugs on resting QEEG activity needs further investigation in longitudinal studies, and it is possible that long-term effects of treatment is a confounding factor when interpreting our current results.

Important considerations of statistical power need to be acknowledged. Calculations of effect sizes are hampered by the few studies available looking at populations at-risk of developing psychosis. Deficits in such populations are likely to be subtler than those in chronic patients. This has been shown to be true for, for example, the P300 event related potential (ERP) peak amplitude (Bramon et al., 2005) and the error-related negativity ERP (Simmonite et al., 2012), and electrophysiological measures of cortical inhibition (Hasan et al., 2012). Furthermore, only a minority of individuals with an at-risk mental state will go on to develop psychosis (Simon et al., 2011; Fusar-Poli et al., 2012; Morrison et al., 2012), making abnormalities in this population difficult to detect. This was clearly observed in a study by Bodatsch et al. (2011) where only at-risk individuals who later converted to psychosis showed EEG abnormalities compared to healthy controls, whereas, similarly to our findings, the overall at-risk group did not differ from controls. Hence, it may be assumed that effect sizes for possible resting EEG abnormalities in at-risk populations are smaller than those in patients. This, in turn, suggests that the current study might have been underpowered to detect true yet subtle differences between healthy controls and at-risk groups.

In conclusion, we set out to characterise resting EEG oscillations (QEEG) in psychosis and populations at risk for this disease and particularly, whether such measures could act as candidate endophenotypes for the illness. Our results provide evidence that chronic psychotic patients exhibit resting QEEG abnormalities in low frequencies. However, no abnormalities were observed in first episode patients or at-risk populations, suggesting that resting QEEG activity is not likely related to genetic risk for the illness. Instead, abnormalities observed in chronic patients may be related to the illness process, or to long-term effects of treatment. Hence, results from this study indicate that resting QEEG activity is not an appropriate candidate endophenotype for genetic research in psychosis, although low frequency activity could be a potential biomarker for non-genetic research, for example as prognostic or medication-response predictors.

## Role of funding source

E. Bramon currently holds a MRC New Investigator Award and a MRC Centenary Award. E. Bramon was further supported by fellowships from the National Institute of Health Research UK and from The Wellcome Trust and by two NARSAD Young Investigator Awards. This research was further funded by The Wellcome Trust, The Psychiatry Research Trust, the Schizophrenia Research Fund, the Brain and Behavior Research Foundation and the NIHR Biomedical Research Centre for Mental Health at the South London and Maudsley NHS Foundation Trust and Institute of Psychiatry Kings College London.

## Contributors

S. Ranlund conducted the statistical analyses, interpreted the analyses, carried out the literature review and wrote the manuscript. J. Nottage conducted the EEG signal processing. M. Shaikh, A. Dutt, and M. Constante contributed to recruitment of participants and data collection. M. Walshe collected data and developed and managed the study database. M-H Hall supervised the study and contributed to recruitment and data collection. K. Friston reviewed the manuscript. R. Murray supervised the study design, facilitated recruitment of participants and data collection. E. Bramon designed the study, collected data and from inception reviewed the manuscript, statistics and literature review. All authors contributed to and have approved the final manuscript.

## Conflict of interest

None of the authors declare any conflicts of interest.

## Figures and Tables

**Table 1:** Sample demographics (N = 279).

	Chronic patients	First episode patients	ARMS	Relatives	Controls
*N* (% of total sample)	48 (17.2%)	46 (16.5%)	33 (11.8%)	45 (16.1%)	107 (38.4%)
*Age* (mean years ± SD)	41.8 ± 11.3	25.0 ± 3.9	23.8 ± 4.0	48.8 ± 16.1	31.6 ± 13.3
Statistics (p value)^a^	t = − 4.6 (< 0.001)	t = 4.7 (< 0.001)	t = 5.4 (< 0.001)	t = − 6.3 (< 0.001)	
*Gender* (% male, male/female)	64.6% (31/17)	69.6% (32/14)	60.6% (20/13)	44.4% (20/25)	51.4% (55/52)
Statistics (p value)^a^	χ^2^ = 2.3 (0.16)	χ^2^ = 4.3 (0.05)	χ^2^ = 0.9 (0.43)	χ^2^ = 0.6 (0.48)	

*Diagnoses* (N, % of group)					
Schizophrenia	33 (68.8%)	12 (26.1%)	–	–	–
Schizoaffective disorder	8 (16.7%)	1 (2.2%)	–	–	–
Brief psychotic disorder	1 (2.1%)	–	–	–	–
Schizophreniform psychosis	–	26 (56.5%)	–	–	–
Bipolar I Disorder	5 (10.4%)	4 (8.7%)	–	–	–
Psychotic disorder NOS	1 (2.1%)	3 (6.5%)	–	–	–
“At risk mental state”^b^	–	–	33 (100.0%)	–	–
Non-psychotic depressive illness (incl. MDD)	–	–	9 (27.3%)^c^	17 (37.8%)	7 (6.5%)
Anxiety disorder (incl. GAD)	–	–	3 (9.1%)^c^	5 (11.1%)	–
Substance Abuse	–	–	4 (12.1%)^c^	–	1 (0.1%)
Personality Disorder	–	–	2 (6.1%)^c^	–	–
No psychiatric illness	–	–	–	23 (51.1%)	99 (92.5%)

*Medication* (N, % of group)^d^					
No psychotropic medication	5 (10.4%)	6 (17.1%)	33 (100%)	45 (100%)	107 (100%)
Amisulpiride	5 (10.4%)	1 (2.9%)	–	–	–
Aripiprazole	4 (8.3%)	5 (14.3%)	–	–	–
Clozapine	7 (14.6%)	–	–	–	–
Flupentixol	4 (8.3%)	–	–	–	–
Olanzapine	14 (29.2%)	10 (28.6%)	–	–	–
Quetiapine	3 (6.3%)	1 (2.9%)	–	–	–
Risperidone	5 (10.4%)	11 (31.4%)	–	–	–
Other antipsychotic	9 (18.8%)	1 (2.9%)	–	–	–
Lithium or Sodium Valproate	9 (18.8%)	6 (17.1%)	–	–	–
Antidepressant	17 (35.4)	4 (11.4%)			
*Years in education* (M ± SD)^e^	12.9 ± 2.2	14.4 ± 2.9	14.1 ± 3.1	12.5 ± 2.2	14.4 ± 2.6

*Ethnicity* (N, % of group)					
Caucasian	44 (91.7%)	8 (17.4%)	20 (60.6%)	43 (95.6%)	76 (71.0%)
African/Caribbean	2 (4.2%)	30 (65.2%)	8 (24.2%)	1 (2.2%)	25 (23.5%)
Other/Mixed	2 (4.2%)	8 (17.4%)	5 (15.2%)	1 (2.2%)	6 (5.6%)

*EEG lab* (N)					
A (64 channels)	–	–	33	–	45
B (40 channels)	48	46	–	45	62

ARMS = At risk mental state; MDD = Major Depressive Disorder; GAD = Generalised Anxiety Disorder;

a 2-tailed t-tests for age and chi square tests for gender, each group compared against the control group;

b ARMS criteria: 67% attenuated psychotic symptoms, 10% brief limited intermittent psychotic symptoms (BLIPS), 10% BLIPS and attenuated symptoms, 3% genetic risk with a decline in function, 10% genetic risk with a decline in function and attenuated symptoms;

c These individuals had a history of a non-psychotic illness in addition to an “at-risk mental state”;

d Data available for 76.1% of first episode group, percentage of 35 first episode patients with information available has been reported.

e Data available for 78.9% of the total sample.

**Table 2:** Average resting EEG amplitude across FZ, CZ and PZ (micro volts ± standard deviations) for all participant groups and frequency bands, uncorrected for covariates.

	Chronic patients	First episode patients	ARMS	Relatives	Controls
Delta	9.03 ± 2.63	8.08 ± 2.34	8.29 ± 2.05	7.17 ± 1.65	8.00 ± 1.94
Theta	12.10 ± 5.28	9.57 ± 3.72	9.38 ± 3.29	8.49 ± 3.24	8.95 ± 2.81
Alpha	8.57 ± 3.04	8.78 ± 4.44	8.60 ± 5.06	7.51 ± 3.84	8.95 ± 4.13
Beta	11.73 ± 3.49	9.21 ± 3.30	10.23 ± 3.65	11.23 ± 5.46	10.56 ± 3.35

ARMS = At risk mental state

**Table 3:** Summary of main significant findings, after correction for multiple testing (p = 0.05/4 = 0.0125).

Delta frequency band	Theta frequency band	Alpha frequency band	Beta frequency band
1.90–3.90 Hz	4.39–7.32 Hz	8.30–12.70 Hz	13.20–21.00 Hz

Chronic patients > controls	Chronic patients > controls	*No significant group differences*	*No significant group differences*
