# Supplementary material for: Resting EEG in psychosis and at-risk populations — A possible endophenotype?
Source: Schizophr Res. 2014 Mar;153(1-3):96–102. doi: 10.1016/j.schres.2013.12.017 (PMC3969576; doi:10.1016/j.schres.2013.12.017)
Supplement: Supplementary file 1 — Supplementary material. [file mmc1.docx]

**Supplementary Material**

1. ***Correlations between outcome variables***

| *Table S1*. Pearson’s correlations between EEG amplitude (log transformed μV) in the four frequency bands and the three scalp sites. | | | | | | | | | | | | | | | | |
| --- | --- | --- | --- | --- | --- | --- | --- | --- | --- | --- | --- | --- | --- | --- | --- | --- |
|  |  | **Delta** | | | **Theta** | | | | **Alpha** | | | | **Beta** | | | |
|  |  | FZ | CZ | PZ | FZ | CZ | PZ | Mean* | FZ | CZ | PZ | Mean* | FZ | CZ | PZ | Mean* |
| **Delta** | FZ |  | 0.90 | 0.52 | 0.73 | 0.70 | 0.46 |  | 0.43 | 0.44 | 0.31 |  | 0.36 | 0.35 | 0.23 |  |
|  | CZ |  |  | 0.59 | 0.73 | 0.79 | 0.53 |  | 0.41 | 0.48 | 0.35 |  | 0.39 | 0.45 | 0.28 |  |
|  | PZ |  |  |  | 0.42 | 0.46 | 0.90 |  | 0.21 | 0.27 | 0.69 |  | 0.28 | 0.31 | 0.74 |  |
| Mean* | |  |  |  |  |  |  | 0.80 |  |  |  | 0.51 |  |  |  | 0.50 |
| **Theta** | FZ |  |  |  |  | 0.96 | 0.60 |  | 0.58 | 0.57 | 0.39 |  | 0.52 | 0.50 | 0.28 |  |
|  | CZ |  |  |  |  |  | 0.65 |  | 0.57 | 0.60 | 0.41 |  | 0.52 | 0.55 | 0.31 |  |
|  | PZ |  |  |  |  |  |  |  | 0.37 | 0.40 | 0.74 |  | 0.38 | 0.40 | 0.76 |  |
| Mean* | |  |  |  |  |  |  |  |  |  |  | 0.62 |  |  |  | 0.58 |
| **Alpha** | FZ |  |  |  |  |  |  |  |  | 0.96 | 0.67 |  | 0.61 | 0.57 | 0.37 |  |
|  | CZ |  |  |  |  |  |  |  |  |  | 0.72 |  | 0.61 | 0.63 | 0.43 |  |
|  | PZ |  |  |  |  |  |  |  |  |  |  |  | 0.45 | 0.47 | 0.78 |  |
| Mean* | |  |  |  |  |  |  |  |  |  |  |  |  |  |  | 0.66 |
| **Beta** | FZ |  |  |  |  |  |  |  |  |  |  |  |  | 0.95 | 0.65 |  |
|  | CZ |  |  |  |  |  |  |  |  |  |  |  |  |  | 0.68 |  |
|  | PZ |  |  |  |  |  |  |  |  |  |  |  |  |  |  |  |
| All correlations significant at the 0.01 level (2-tailed).  * Correlations between mean log-transformed amplitudes (μV) in the frequency bands across FZ, CZ, and PZ. | | | | | | | | | | | | | | | | |

EEG amplitude (log transformed μV) in the four frequency bands and the three scalp sites were all significantly correlated (p<0.01), with correlation coefficients ranging between 0.21 and 0.99. Within each frequency band, correlations tended to be stronger between neighbouring electrodes (FZ-CZ or CZ-PZ) and weaker when FZ was compared to PZ. At the same location, delta and theta as well as alpha and beta tended to show the strongest correlations.

1. ***Full Statistical Results***

| *Table S2*. Mixed model linear regression results, separate for the four frequency bands | | | | | | |
| --- | --- | --- | --- | --- | --- | --- |
| **Delta frequency band** | | | | | | |
|  | **Coefficient (log10)** | **Standard error** | **z** | **Uncorrected p-value** | **95% confidence interval** | |
| *Controls vs.* |  |  |  |  |  |  |
| Relatives | 0.006 | 0.020 | 0.320 | 0.746 | -0.033 | 0.046 |
| ARMS | -0.011 | 0.022 | -0.490 | 0.621 | -0.053 | 0.032 |
| First episodes | 0.005 | 0.020 | 0.260 | 0.799 | -0.034 | 0.044 |
| Chronics | 0.082 | 0.019 | 4.350 | <0.001 | 0.046 | 0.118 |
| *FZ vs.* |  |  |  |  |  |  |
| CZ | -0.011 | 0.004 | -2.940 | 0.003 | -0.018 | -0.004 |
| PZ | -0.055 | 0.004 | -15.330 | <0.001 | -0.062 | -0.048 |
| *Covariates* |  |  |  |  |  |  |
| Age | -0.002 | 0.001 | -3.850 | <0.001 | -0.003 | -0.001 |
| Gender | 0.042 | 0.012 | 3.550 | <0.001 | 0.019 | 0.065 |
| Lab | 0.022 | 0.020 | 1.100 | 0.272 | -0.018 | 0.062 |
| Constant | 0.976 | 0.040 | 24.640 | <0.001 | 0.898 | 1.054 |
| **Theta frequency band** | | | | | | |
|  | **Coefficient (log10)** | **Standard error** | **z** | **Uncorrected p-value** | **95% confidence interval** | |
|  |  |  |  |  |  |  |
| *Controls vs.* |  |  |  |  |  |  |
| Relatives | 0.014 | 0.029 | 0.470 | 0.637 | -0.044 | 0.072 |
| ARMS | 0.004 | 0.031 | 0.140 | 0.891 | -0.056 | 0.064 |
| First episodes | 0.012 | 0.028 | 0.410 | 0.679 | -0.044 | 0.067 |
| Chronics | 0.136 | 0.027 | 4.980 | <0.001 | 0.083 | 0.190 |
| *FZ vs.* |  |  |  |  |  |  |
| CZ | -0.009 | 0.004 | -2.640 | 0.008 | -0.016 | -0.002 |
| PZ | -0.063 | 0.004 | -17.450 | <0.001 | -0.070 | -0.056 |
| *Covariates* |  |  |  |  |  |  |
| Age | -0.002 | 0.001 | -3.000 | 0.003 | -0.004 | -0.001 |
| Gender | 0.033 | 0.016 | 2.040 | 0.041 | 0.001 | 0.065 |
| Lab | 0.002 | 0.029 | -0.070 | 0.945 | -0.059 | 0.055 |
| Constant | 1.057 | 0.056 | 18.810 | <0.001 | 0.947 | 1.167 |
| **Alpha frequency band** | | | | | | |
|  | **Coefficient (log10)** | **Standard error** | **z** | **Uncorrected p-value** | **95% confidence interval** | |
| *Controls vs.* |  |  |  |  |  |  |
| Relatives | -0.026 | 0.038 | -0.700 | 0.486 | -0.100 | 0.048 |
| ARMS | -0.045 | 0.039 | -1.140 | 0.254 | -0.122 | 0.032 |
| First episodes | 0.008 | 0.036 | 0.220 | 0.829 | -0.063 | 0.079 |
| Chronics | 0.035 | 0.035 | 1.000 | 0.319 | -0.034 | 0.104 |
| *FZ vs.* |  |  |  |  |  |  |
| CZ | 0.018 | 0.005 | 3.770 | <0.001 | 0.009 | 0.027 |
| PZ | 0.060 | 0.005 | 12.610 | <0.001 | 0.051 | 0.069 |
| *Covariates* |  |  |  |  |  |  |
| Age | -0.001 | 0.001 | -1.510 | 0.132 | -0.003 | <0.001 |
| Gender | 0.058 | 0.021 | 2.780 | 0.005 | 0.017 | 0.098 |
| Lab | 0.031 | 0.037 | 0.820 | 0.411 | -0.042 | 0.104 |
| Constant | 0.903 | 0.072 | 12.570 | <0.001 | 0.762 | 1.044 |
| **Beta frequency band** | | | | | | |
|  | **Coefficient (log10)** | **Standard error** | **z** | **Uncorrected p-value** | **95% confidence interval** | |
| *Controls vs.* |  |  |  |  |  |  |
| Relatives | 0.034 | 0.028 | 1.190 | 0.232 | -0.022 | 0.089 |
| ARMS | -0.022 | 0.029 | -0.740 | 0.457 | -0.079 | 0.036 |
| First episodes | -0.013 | 0.027 | -0.460 | 0.644 | -0.066 | 0.041 |
| Chronics | 0.062 | 0.026 | 2.360 | 0.018 | 0.010 | 0.113 |
| *FZ vs.* |  |  |  |  |  |  |
| CZ | 0.013 | 0.003 | 3.860 | <0.001 | 0.006 | 0.019 |
| PZ | 0.007 | 0.003 | 2.100 | 0.035 | <0.001 | 0.013 |
| *Covariates* |  |  |  |  |  |  |
| Age | 0.001 | 0.001 | 1.420 | 0.155 | <-0.001 | 0.002 |
| Gender | 0.070 | 0.016 | 4.500 | <0.001 | 0.040 | 0.101 |
| Lab | 0.046 | 0.028 | 1.630 | 0.102 | -0.009 | 0.100 |
| Constant | 0.901 | 0.054 | 16.750 | <0.001 | 0.795 | 1.006 |

*Delta frequency band*: Amplitude was significantly higher in FZ compared to CZ (p=0.003) and PZ (p<0.001) in the control group. Furthermore, in the control group, there were significant effects of age (p<0.001), with amplitude decreasing with age, and gender (p<0.001), with females showing higher amplitudes than males. There was no significant effect of lab.

*Theta frequency band*: Regional effects showed that, in the control group, the amplitude reduced significantly from FZ to CZ (p=0.008) and from FZ to PZ (p<0.001). Further, there were significant effects of age (p=0.003), with theta amplitude reducing with age, and gender (p=0.041), with females showing higher amplitudes than males. There was no significant effect of lab.

*Alpha frequency band*: In the control group, there were significant regional effects, with the amplitude increasing from FZ to CZ (p<0.001) as well as from FZ to PZ (p<0.001). Further, females in the control group showed significantly higher resting alpha amplitudes compared to males (p=0.005). There were no significant effects of age or lab in the alpha frequency band.

*Beta frequency band*: Regional effects showed that, in the control group, the amplitude increased significantly from FZ to CZ (p<0.001) and from FZ to PZ (p=0.035). Females showed significantly greater amplitudes than males in the control group (p<0.001). There were no significant age or lab effects.

1. ***Additional post-hoc analysis: Chronic patients vs. other groups***

| *Table S3*. Mixed model linear regression results using chronic patient group as reference group, separate for the four frequency bands | | | | | | |
| --- | --- | --- | --- | --- | --- | --- |
| **Delta frequency band** | | | | | | |
|  | **Coefficient (log10)** | **Standard error** | **z** | **Uncorrected p-value** | **95% confidence interval** | |
| *Chronics vs.* |  |  |  |  |  |  |
| Controls | -0.08 | 0.02 | -4.35 | <0.001 | -0.12 | -0.05 |
| Relatives | -0.08 | 0.03 | -3.91 | <0.001 | -0.11 | -0.04 |
| ARMS | -0.09 | 0.03 | -3.23 | 0.001 | -0.15 | -0.03 |
| First episodes | -0.08 | 0.02 | -3.54 | <0.001 | -0.12 | -0.03 |
| **Theta frequency band** | | | | | | |
|  | **Coefficient (log10)** | **Standard error** | **z** | **Uncorrected p-value** | **95% confidence interval** | |
|  |  |  |  |  |  |  |
| *Chronics vs.* |  |  |  |  |  |  |
| Controls | -0.14 | 0.03 | -4.98 | <0.001 | -0.19 | -0.08 |
| Relatives | -0.12 | 0.02 | -4.96 | <0.001 | -0.17 | -0.07 |
| ARMS | -0.13 | 0.04 | -3.22 | 0.001 | -0.21 | -0.05 |
| First episodes | -0.12 | 0.03 | -4.03 | <0.001 | -0.18 | -0.06 |
| **Alpha frequency band** | | | | | | |
|  | **Coefficient (log10)** | **Standard error** | **z** | **Uncorrected p-value** | **95% confidence interval** | |
| *Chronics vs.* |  |  |  |  |  |  |
| Controls | -0.03 | 0.04 | -1.00 | 0.319 | -0.10 | 0.03 |
| Relatives | -0.06 | 0.03 | -1.99 | 0.047 | -0.12 | -0.01 |
| ARMS | -0.08 | 0.05 | -1.52 | 0.129 | -0.18 | 0.02 |
| First episodes | -0.03 | 0.04 | -0.68 | 0.494 | -0.10 | 0.05 |
| **Beta frequency band** | | | | | | |
|  | **Coefficient (log10)** | **Standard error** | **z** | **Uncorrected p-value** | **95% confidence interval** | |
| *Chronics vs.* |  |  |  |  |  |  |
| Controls | -0.06 | 0.026 | -2.36 | 0.018 | -0.11 | -0.01 |
| Relatives | -0.027 | 0.02 | -1.20 | 0.231 | -0.07 | 0.02 |
| ARMS | -0.08 | 0.039 | -2.13 | 0.032 | -0.16 | -0.01 |
| First episodes | -0.07 | 0.029 | -2.51 | 0.012 | -0.13 | -0.02 |
| Bonferroni correction for 4 tests gives a significance threshold of p = 0.05 / 4 = 0.0125 | | | | | | |

*Delta frequency band:* The chronic patient group showed significantly increased resting EEG activity compared to all other groups (all p=0.001 or less). All such comparisons remained significant after correction for multiple testing.

*Theta frequency band:* Again, the chronic patient group showed significantly increased activity compared to the other groups, surviving multiple testing (all p=0.001 or less).

*Alpha frequency band:* No group differed significantly from the chronic patient group.

*Beta frequency band:* The chronic patients showed increased resting EEG activity compared to healthy controls and first episode patients. The former comparison did not survive correction for multiple testing (uncorrected p=0.018), but the difference between chronic and first episode patients remained significant at a trend level (uncorrected p=0.012).
